# *In Vivo* MRI Assessment of Hepatic and Splenic Disease in a Murine Model of Schistosmiasis

**DOI:** 10.1371/journal.pntd.0004036

**Published:** 2015-09-22

**Authors:** Brice Masi, Teodora-Adriana Perles-Barbacaru, Caroline Laprie, Helia Dessein, Monique Bernard, Alain Dessein, Angèle Viola

**Affiliations:** 1 Génétique et Immunologie des Maladies Parasitaires-Unité Mixte de Recherche S_906, Aix-Marseille Université, Marseille, France; 2 Unité 906, Institut National de la Santé et de la Recherche Médicale, Marseille, France; 3 Centre de Résonance Magnétique Biologique et Médicale-Unité Mixte de Recherche 7339, Aix-Marseille Université, Marseille, France; 4 Unité Mixte de Recherche 7339, Centre National de la Recherche Scientifique, Marseille, France; 5 Laboratoire VET-HISTO, Marseille, France; 6 Laboratoire de Parasitologie-Mycologie, Centre Hospitalier Universitaire Timone, Assistance Publique des Hôpitaux de Marseille, Marseille, France; Uniformed Services University, UNITED STATES

## Abstract

**Background:**

Schistosomiasis (or bilharzia), a major parasitic disease, affects more than 260 million people worldwide. In chronic cases of intestinal schistosomiasis caused by trematodes of the *Schistosoma genus*, hepatic fibrosis develops as a host immune response to the helminth eggs, followed by potentially lethal portal hypertension. In this study, we characterized hepatic and splenic features of a murine model of intestinal schistosomiasis using *in vivo* magnetic resonance imaging (MRI) and evaluated the transverse relaxation time T_2_ as a non-invasive imaging biomarker for monitoring hepatic fibrogenesis.

**Methodology/Principal Findings:**

CBA/J mice were imaged at 11.75T two, six and ten weeks after percutaneous infection with *Schistosoma mansoni*. *In vivo* imaging studies were completed with histology at the last two time points. Anatomical MRI allowed detection of typical manifestations of the intestinal disease such as significant hepato- and splenomegaly, and dilation of the portal vein as early as six weeks, with further aggravation at 10 weeks after infection. Liver multifocal lesions observed by MRI in infected animals at 10 weeks post infection corresponded to granulomatous inflammation and intergranulomatous fibrosis with METAVIR scores up to A2F2. While most healthy hepatic tissue showed T_2_ values below 14 ms, these lesions were characterized by a T_2_ greater than 16 ms. The area fraction of increased T_2_ correlated (r_S_ = 0.83) with the area fraction of Sirius Red stained collagen in histological sections. A continuous liver T_2_* decrease was also measured while brown pigments in macrophages were detected at histology. These findings suggest accumulation of hematin in infected livers.

**Conclusions/Significance:**

Our multiparametric MRI approach confirms that this murine model replicates hepatic and splenic manifestations of human intestinal schistosomiasis. Quantitative T_2_ mapping proved sensitive to assess liver fibrogenesis non-invasively and may therefore constitute an objective imaging biomarker for treatment monitoring in diseases involving hepatic fibrosis.

## Author Summary

Schistosomiasis (or bilharzia), a major helminth disease, affects more than 260 million people worldwide. While the adult worms survive for years within veins of the gastrointestinal system, symptoms are due to inflammatory reactions to their eggs in several organs. Hepatic fibrosis may develop in chronic cases of infection with *Schistosoma mansoni* and lead to portal hypertension with potentially lethal complications. In this study, we aimed at establishing a non-invasive quantitative and readily available magnetic resonance imaging (MRI) technique to monitor *in vivo* the development of hepatic fibrosis and portal hypertension in *Schistosoma mansoni* infected mice. We evaluated the transverse relaxation time T_2_, an easily measurable MRI parameter, as an early and quantitative imaging biomarker for hepatic fibrogenesis and validated it with histological techniques for fibrosis detection and quantification. In addition, we confirmed that this mouse model of schistosomiasis replicates the human pathology closely. The quantitative imaging biomarkers validated in this study will aid in the preclinical and clinical evaluation of new therapeutic strategies against hepatic fibrogenesis.

## Introduction

Present in many tropical and subtropical countries, schistosomiasis (or bilharzia), the second most prevalent parasitic disease in the world after malaria, affects more than 260 million people and leads to 200 000 deaths per year [1]. This helminthic disease is caused by trematodes of the *Schistosoma* genus. *S*. *haematobium*, *S*. *mansoni*, and *S*. *japonicum* are the main species infecting humans. These parasites have aquatic gastropods as intermediate hosts and a final vertebrate host. *S*. *mansoni* is the principal agent of digestive forms of the human disease. The gastropod is infected with *miracidia* released from the *S*. *mansoni* eggs that transform into sporocysts. These sporocysts shed *cercariae* in the water that can penetrate the skin of the mammalian hosts. After maturation, male and female worms reproduce in the mesenteric venous plexus and produce eggs that are discharged with the stool into the environment [2,3,4].

However, many eggs are not eliminated and disseminate in the intestines and the liver where they obstruct presinusoidal capillary venules [2,4,5]. The host immune response elicited by these eggs leads to the formation of periovular granulomas and tissue damage. An imbalance between scarring and regeneration causes an accumulation of extracellular matrix rich in collagen (particularly types I and III) leading to hepatic fibrosis [6]. Hepatomegaly occurs early in the disease as a consequence of the granulomatous inflammation [2,3]. In *ca* 10% of patients, 5–20 years after infection, serious complications occur such as splenomegaly, pulmonary arterial hypertension accompanied by right heart failure, periportal fibrosis resulting in portal hypertension and esophageal varices, that can lead to ascites and gastrointestinal bleeding with high mortality [3,4,5,7,8]. Chronic schistosomiasis is also associated with an increased incidence of hepatocellular carcinoma [4,9]. The standard treatment is the trematodicide Praziquantel, which kills adult worms, but is ineffective on juvenile mammalian-stage schistosomes. The mechanism of action and molecular targets of Praziquantel are unknown. However many studies have reported vacuolation and blebbing of worm teguments and suggested a direct disruptive effect on Ca^2+^ channels, whereas a recent work has described distinct effects on male and female worms [10,11]. Although effective even at a single dose, reinfection is frequent and in 2013 only 13% of people necessitating treatment could benefit from it [1]. Despite extensive research, an anti-schistosomiasis vaccine is not yet available [12,13].

The mouse model of schistosomiasis is well suited for pharmaceutical and basic research purposes since mice are infected with the parasite species that are pathogenic for humans, and are therefore expected to accurately recapitulate the pathological features of the human disease. The objective of this study was to provide the first characterization of hepatic and splenic features of murine intestinal schistosomiasis using *in vivo* magnetic resonance imaging (MRI). Another aim was to search for non-invasive biomarkers allowing future evaluation of new therapeutic strategies against the intestinal form of the disease in murine models.

Ultrasound is the leading medical imaging examination for the diagnosis of human intestinal schistosomiasis, and allows the assessment of liver involvement and portal hypertension [14,15]. Liver fibrosis can be monitored using ultrasound transient elastography (FibroScan), a technique allowing the assessment of liver stiffness [16]. Elastography is based on the measurement of tissue elasticity following the propagation of a mechanical shear wave through the liver. However, ultrasound transient elastography has the disadvantage of being highly operator dependent, less reliable for deep organs and not readily available for the exploration of rodent models [17]. Analysis of texture features from computed tomography (CT) images enables staging of fibrosis throughout the liver, but is less accurate in case of heterogeneous fibrosis distribution and is considered inferior to ultrasound transient elastography [18]. Whole abdominal coverage can also be achieved with MRI. In addition to the detection of morphological indicators of liver fibrosis such as splenomegaly and portal hypertension on conventional images [19], various MRI methods can be used for the study of (schistosomiasis-induced) liver disease. Magnetic resonance elastography (MRE) measures viscoelastic properties of the liver tissue, namely its capacity to return with time to its original shape after the application of deforming forces, by quantifying the propagation of the shear waves. This technique can be implemented on standard MRI systems but requires a mechanical vibrator device for the generation of shear waves. Liver stiffness has been shown to correlate with fibrosis stage in patients [20] and animal models [21]. A recent study suggests that MRE more accurately discriminates between early fibrosis stages than ultrasound transient elastography [22]. However, increased liver stiffness is not specific for fibrosis [23], and MRE can be hampered in subjects with severe iron overload [22]. Qualitative and quantitative scores of liver texture features on double contrast-enhanced MRI, an MRI technique based on the injection of two different types of contrast agents (super paramagnetic iron oxides and gadolinium chelates) have been shown to distinguish between mild and severe fibrosis [24]. In rodent models, MRI using a collagen type I targeted gadolinium-based contrast agent has shown differential uptake and washout in fibrotic livers with a good correlation to histological quantification of collagen [25,26]. Diffusion-weighted MRI, a technique sensitive to water diffusivity generally used to study tissue microstructure, has also been performed since fibrosis is expected to restrict water motion [27]. However, due to lack of standardization and confounding factors such as altered perfusion or accompanying inflammation this technique is not sensitive to mild stages of liver fibrosis [28,29]. Another MRI technique, known as intravoxel incoherent motion imaging [30] can distinguish between microscopic motion of water molecules in intra- and extracellular compartments and the microcirculation of blood. Consequently, the derived parameters correlate better with fibrosis stage than conventional diffusion-weighted imaging in patients [31] and animal models [32]. However, this technique has not proved superior to MRE in distinguishing mild to intermediate fibrosis stages in patients [33]. Relaxometry has been proposed for the staging of hepatic fibrosis and evaluated in a number of studies. Relaxometry studies magnetic relaxation, a process by which magnetization of magnetic nuclei returns to its equilibrium state (parallel to the static magnetic field, B_0_) after it was disturbed from equilibrium and tipped into the plane orthogonal to B_0_ (transverse plane) by a radiofrequency pulse. Two relaxation time constants can be measured: the longitudinal relaxation time-constant T_1_ corresponding to the recovery of the magnetization along B_0,_ and the transverse relaxation time-constant T_2_ describing the decay of the magnetization in the transverse plane. Another relaxation time, called T_2_*, is a measure of T_2_ taking into account the effects of static magnetic field inhomogenities on relaxation. Relaxation time constants depend on tissue structure and composition, which may vary with physiological or pathological processes, and strongly influence contrast in MRI. Maps derived from MRI images representing a spatial distribution of quantitative values of a selected relaxation time-constant (T_2_, T_2_* or T_1_) can be generated. Although relaxation time-constant values may vary with magnetic field strength, the tendency to increase or decrease upon a physiological or pathological process is independent of the magnetic field strength. An increase of the longitudinal relaxation time T_1_ has been observed with increasing severity of hepatic fibrosis. However, alterations of the transverse relaxation time T_2_ during the process of hepatic fibrogenesis were generally more sensitive [34,35,36]. In particular, in pre-clinical MRI studies using MRI systems equipped with high or ultra high magnetic field strength (3T < B_0_ < 21T) to increase the spatial resolution or the signal-to-noise ratio, T_1_ relaxation times tend to differ less between tissues than transverse relaxation times. In addition, T_2_ mapping is less challenging than T_1_ mapping in conjunction with respiratory gating (synchronization of MRI sequences with respiration) since all echoes required for T_2_ analysis are acquired within a few hundred milliseconds, while T_1_ mapping requires repeated sampling over a couple of seconds. The T_2_ and T_2_* relaxation times are also sensitive markers of iron deposition and hemorrhage [37,38].

In this study, multiparametric MRI including quantitative T_2_ and T_2_* mapping was performed in an attempt to establish a quantitative measure of the liver damage and related complications caused by schistosomiasis, and in particular to assess early stages of liver fibrosis. A mouse model of the intestinal form of the disease obtained with *S*. *mansoni* was explored. *In vivo* anatomical and relaxometry findings were compared to histological staging of liver disease and fibrosis progression.

## Materials and Methods

### Ethics statement

Animal studies were in agreement with the French guidelines for animal care from the French Ministry for Agriculture (Animal Rights Division), the directive 2010/63/EU of the European Parliament and of the Council of 22 September 2010, and approved by our institutional committee on Ethics in animal research (Comité d’Ethique de Marseille n°14, project authorization n°: 02157.02).

### Animals and schistosomiasis induction

Twenty-four female CBA/J mice (6-week old) from Charles River Laboratories (l’Arbresle, France) were used. Mice were maintained at 22.5°C with a 12h light/12h dark cycle in an enriched environment with free access to food and water. Twelve mice were infected percutaneously at the age of seven weeks with 30 *cercariae* of the Venezuelan strain of *S*. *mansoni* under intraperitoneal anesthesia (ketamine 100 mg/kg, xylazine 4 mg/kg). *Cercariae*, which are maintained in our laboratory by passage through *Biomphalaria glabrata* snails, were counted under binocular microscope, diluted in 500 μl of water, and placed for 60 minutes on the sheared abdomen of mice to replicate the natural route of infection.

Mice were weighed before each MRI session. *In vivo* MRI was performed on two animal cohorts. The first cohort included a group of 6 infected mice and a group of 6 uninfected mice, which were both imaged at 2 weeks and 6 weeks post infection. MRI at 6 weeks was followed by histology. The second cohort consisting of a group of 6 infected mice and a group of 6 uninfected mice was explored only once at 10 weeks post infection and the animals were sacrificed after MRI for histology.

### *In vivo* MRI protocol

MRI experiments were performed on a Bruker AVANCE 500 WB MR system (Bruker, Ettlingen, Germany) operating at very high magnetic field (11.75 T), equipped with actively shielded gradients (1 T/m maximum gradient strength and 9 kT/m/s maximum slew rate) and a 30 mm-diameter transmitter/receiver volume birdcage coil. A catheter was inserted into the intraperitoneal cavity of the mice for contrast agent delivery. The animals were positioned in a cradle, and a pneumatic pressure probe was placed under their chest for respiration monitoring. Anesthesia was maintained with isoflurane in air using 1.3–1.8% *via* a face mask and body temperature was maintained using the water circulation of the gradient cooling system set to 42°C.

All sequences were prospectively gated with respiration using an MRI compatible monitoring and gating system (PC-SAM, Small Animal Instruments Inc., Stony Brook, NY). Images were acquired in the transverse plane with a field of view of 24×24 mm^2^ and a slice thickness of 0.5 mm. Structural imaging at high in plane resolution (matrix 240×240, in-plane resolution 100 μm) was performed using a 2D spin-echo sequence (repetition time TR ≥ 448 ms; echo time TE = 14 ms) with 20 contiguous slices and repeated on adjacent 20 slices to cover the liver and spleen entirely. Images were acquired before and 15 minutes after intraperitoneal injection of a paramagnetic contrast agent (50 μl of 0.5 M gadoteric acid, DOTAREM, Guerbet, Villepinte, France) (number of accumulations for each acquisition: 2 and 4 respectively). Prior to contrast agent injection, T_2_ and T_2_* maps (TR ≥ 9 s; matrix 64×64, 2 accumulations) were acquired in a single slice positioned 0.5 mm caudal of the bifurcation of the portal vein, using a multi-spin echo sequence (12 equally spaced echoes at TE = 7.5 to 120 ms) and a multi-gradient echo sequence (8 equally spaced echoes at TE = 1.6 to 13.5 ms), respectively. Using the bifurcation of the portal vein as landmark this axial slice position was reproducible between animals and covered sufficient liver tissue. Respiratory rate was kept between 60 and 70 breaths per minute by adjusting the isoflurane percentage leading to a total acquisition time of approximately 15 min for the anatomical imaging and 20 min for each map. For quality control, an external reference with a known T_2_ of *ca* 21 ms, consisting of a capillary filled with the paramagnetic contrast agent diluted in saline was placed in the image field of view.

### MRI relaxometry of collagen solutions

Relaxometry studies were performed at 11.75T on water and solutions of collagen at four different concentrations (1.66 g/L, 3.33 g/L, 6.25 g/L and 12.5 g/L). Collagen solutions were prepared with Type I collagen from rat tail (Sigma-Aldrich, St Quentin Fallavier, France) solubilized under magnetic stirring in 0.1 M acetic acid at 40°C. T_1_ and T_2_ relaxation times were measured using an inversion recovery gradient echo sequence with 7 inversion times (T_inv_ 15 ms to 15 s) and a multi-spin echo sequence with 80 TE (10 to 800 ms). TR was 20 s.

### MRI processing

Images were analyzed under ImageJ (Rasband, W.S., ImageJ, U. S. National Institutes of Health, Bethesda, Maryland, USA, http://imagej.nih.gov/ij/, 1997–2014, last access January 2015) for liver and spleen volumetry, portal vein diameter, and T_2_-map generation.

Liver and spleen were delineated on each slice (i) to measure the cross-section (A_i_) of the organ and estimate its volume in mm^3^ by $V=0.5\sum_{i}A_{i}$. To assess portal hypertension, the portal vein cross-section was measured 0.5 mm caudal of the portal bifurcation, and its diameter was estimated as the Feret’s diameter. The Feret’s diameter is the maximum caliper length namely the longest distance between any two points along the section boundary. Relaxation time maps were computed by fitting the signal intensity S to $S(TE)=S_{0}\exp(TE/T_{2}^{\left(*\right)})$ or *S*(*T*_*inv*_) = *S*_0_|1 − 2*E* exp(−*T*_*inv*_/*T*_1_)| using the simplex algorithm in ImageJ. Here, *S*_*0*_ is the signal at thermal equilibrium, and *E* the efficiency of the inversion pulse. Histograms of quantitative T_2_ and T_2_* were obtained from a large region of interest (ROI) covering the liver but excluding the hepatic hilus and large vessels as well as the gall bladder, bowels or stomach when present in the slice.

### Histology

After euthanasia, livers were fixed in 10% buffered formalin for a minimum of 48 hours, sampled according to standardized procedures [39], paraffin-embedded and routinely processed for histology. Three 3-μm paraffin serial sections per mouse were stained with Hematoxylin-Eosin (HE), with Sirius Red for collagen, and Perls’ stain for iron. Grading of inflammatory activity and staging of fibrosis was performed according to the METAVIR scoring system, a histological scale used to quantify the degree of inflammation and fibrosis of a liver biopsy. “A” refers to the intensity of necrosis and inflammation and may vary from A0 to A3 (A0 = no activity, A1 = mild activity, A2 = moderate activity, and A3 = severe activity). “F” refers to the extent of fibrosis and may vary from F0 to F4 (F0 = no fibrosis, F1 = portal fibrosis without septa, F2 = portal fibrosis with rare septa, F3 = numerous septa without cirrhosis, and F4 = cirrhosis) [40]. Sirius Red stained sections were examined by semiautomatic computer-based morphometry using the NIKON DS-Fi2 camera and NIS Elements imaging software (Nikon, Japan). Morphometric analysis was made on one entire lobe section, measuring semi-automatically delineated Sirius Red stained zones (S1A Fig) and subtracting when present the egg area or unstained central granuloma area deprived of collagen deposition. All analyses were performed in a blinded manner.

### Statistics

Statistical analyses were performed using either GraphPad Prism version 5.00 (San Diego, CA) or JMP 9.0 (SAS, Cary, NC). Values are reported as means ± standard deviation. The non-parametric Mann-Whitney test was used to compare uninfected and infected mice at each time point. The Kruskal-Wallis test was used to compare the METAVIR score or the percentage of Sirius Red stained zones among controls and diseases mice at 6 and 10 weeks after infection. Correlations between T_2_ values and METAVIR score or T_2_ values and fibrosis quantification by Sirius Red were performed using the Spearman test. The Smirnov test was used to compare distributions from two independent samples [41]. Values of P<0.05 were considered significant.

## Results

### Clinical follow-up

Weight control performed before each MRI session showed a continuous increase without significant difference between infected and control groups at any time point. No signs of general health alteration, such as cachexia, reduced mobility or behavioral changes were observed, suggesting animals were in good health condition during the study. All the animals of the first cohort exposed to the *cercariae* of *S*. *mansoni* were successfully infected as confirmed by MRI and histology at 6 weeks after infection, whereas only 4 out of 6 mice of the second cohort were successfully infected and developed hepatosplenic signs as shown by MRI and histology at 10 weeks after infection.

### *In vivo* MRI

All the images acquired were of good quality, except one T_2_* map obtained from a control animal of the second cohort, which showed motion artefacts and therefore was not included in the analysis. The two mice of the second cohort in which the infection had failed were also discarded from the analysis.

**Fig 1:**
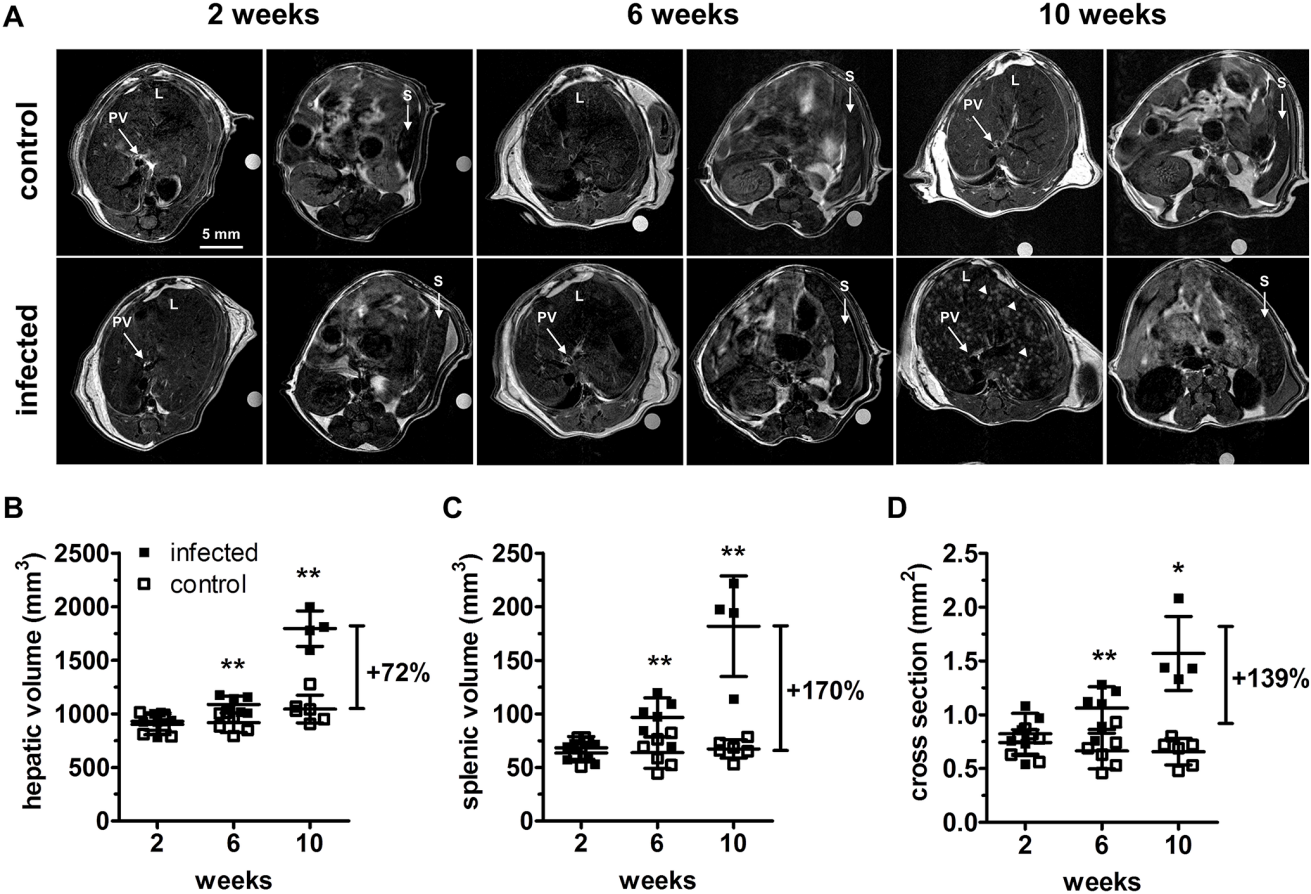
MRI signs of organomegaly and portal hypertension. (**A**) Comparison of contrast-enhanced anatomical MRI covering liver and spleen in a representative infected and a control mouse at 2, 6, and 10 weeks post infection. Two images are shown for each mouse, one centered on the liver and the portal vein (left image for each couple of images), and another one showing the spleen (right image). A multifocal liver and spleen damage is observable at 10 weeks. Spleen (vertical arrows), portal vein (oblique arrows), liver multifocal hyperintensities (arrowheads). The circular object is the external reference tube. Progressive increases in hepatic volume (**B**), spleen volume (**C**) and portal vein cross section (**D**) are detected from week 6 to10 after infection. (*P<0.05, **P<0.01), filled squares = infected animals, empty squares = control animals. Abbreviations: L = liver, PV = portal vein, S = spleen.

**Fig 2:**
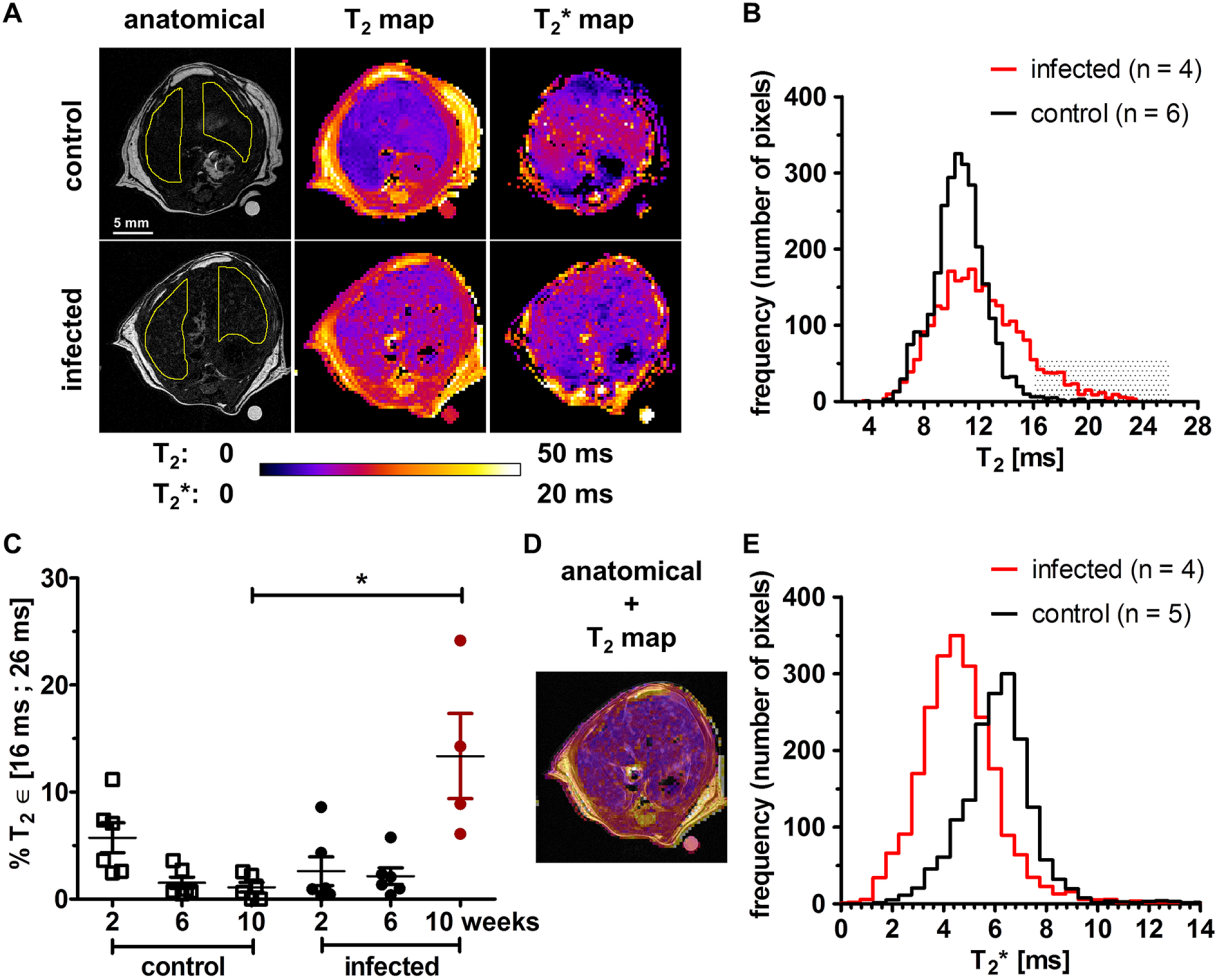
Quantitative T_2_ and T_2_* mapping at 10 weeks post infection. (**A**) Non contrast-enhanced anatomical acquisitions, T_2_ and T_2_* maps for two representative mice from the control and the schistosomiasis group (color bar range: 0 to 50 ms for T_2_ maps, 0 to 20 ms for T_2_* maps). Spatial patterns of increased T_2_ and decreased T_2_* can be observed in the infected mouse. The external reference has an average T_2_ of 21.55 ± 0.25 ms (2 weeks: 21.74±0.22 ms, 6 weeks 21.64 ± 0.65 ms, 10 weeks: 21.26±0.60 ms, P = 0.1139). Regions of interest (ROI) in the liver used for analysis are shown on the anatomical acquisitions. **(B)** At 10 weeks post infection the T_2_ distribution is enlarged (P < 0.01) in infected (n = 4) compared to control livers (n = 6) with a fraction of values exceeding 16 ms. **(C)** The area fraction with T_2_ ϵ [16 ms;26 ms] is significantly increased at 10 weeks post infection compared with control mice (Dunn’s Multiple Comparison Test P < 0.05). The overlay **(D)** shows that hepatic regions with increased T_2_ co-localize with hyperintensities seen in the anatomical images prior to Gd-DOTA injection. These hyperintense lesions further enhance after Gd-DOTA injection, but organ displacement prevents pixelwise comparison with T_2_ maps. (**E)** Distributions of hepatic T_2_* values at 10 weeks post infection showing decreased T_2_* values (P < 0.01) in the infected mice (n = 4) compared to controls (n = 5).

While no differences were detected between infected and control groups at 2 weeks post infection by anatomical MRI (Fig 1A) and relaxometry, anatomical MRI was able to detect signs related to murine schistosomiasis (Fig 1B–1D) as early as 6 weeks post infection. Indeed, hepatomegaly (Fig 1B), splenomegaly (Fig 1C), and portal hypertension (Fig 1D) as assessed by MRI were significant at 6 weeks: +19% (P = 0.0043), +52% (P = 0.0087) and +60% (P = 0.0087) and progressed to +72% (P = 0.0095), +170% (P = 0.0095) and +139% (P = 0.0139) 10 weeks post infection, respectively. The evolution of the portal vein diameter was similar to that of the cross-section (control: 1.19±0.12 mm, infected: 1.25±0.13 mm, P = 0.393 at 2 weeks, control: 1.17±0.19 mm, infected: 1.43±0.16 mm, P = 0.027 at 6 weeks, and control: 1.16±0.10 mm, infected: 1.68±0.13 mm, P = 0.004 at 10 weeks). However, the most remarkable findings were multifocal hyperintensities (disseminated “white spots”) of the liver in 4 infected mice at 10 weeks post infection (Fig 1A). These hyperintensities were visible before contrast agent injection (Fig 2A), and were contrast-enhanced after injection.

The average T_2_ value in hepatic ROIs (Fig 2A) at 10 weeks post infection was not significantly increased compared to the average T_2_ value in control livers (control: 10.4 ± 1.3 ms, infected: 12.2 ± 1.5 ms, P = 0.1714). However, the distributions of hepatic T_2_ values in infected and control mice were different (Fig 2B). The area fraction with 16 ms < T_2_ < 26 ms was 13.4 ± 6.9% in infected animals at 10 weeks, while it was 1.1 ± 1.0% in control mice (Kruskal-Wallis test P = 0.0048) (Fig 2C). Liver T_2_ values > 16 ms on the T_2_ maps were co-localized with the hyperintensities on anatomical images (Fig 2D). The distributions of T_2_* values in the livers of infected mice were shifted to lower values at 6 weeks (average T_2_* = 5.4 ± 1.5 ms in infected mice *versus* T_2_* = 5.8 ± 1.6 ms in control mice) and at 10 weeks (average T_2_* = 4.6 ± 1.5 ms in infected mice *versus* T_2_* = 6.1 ± 1.5 ms in control mice) post infection (Fig 2E).

### Relaxometry of MRI solutions of collagen

**Fig 3:**
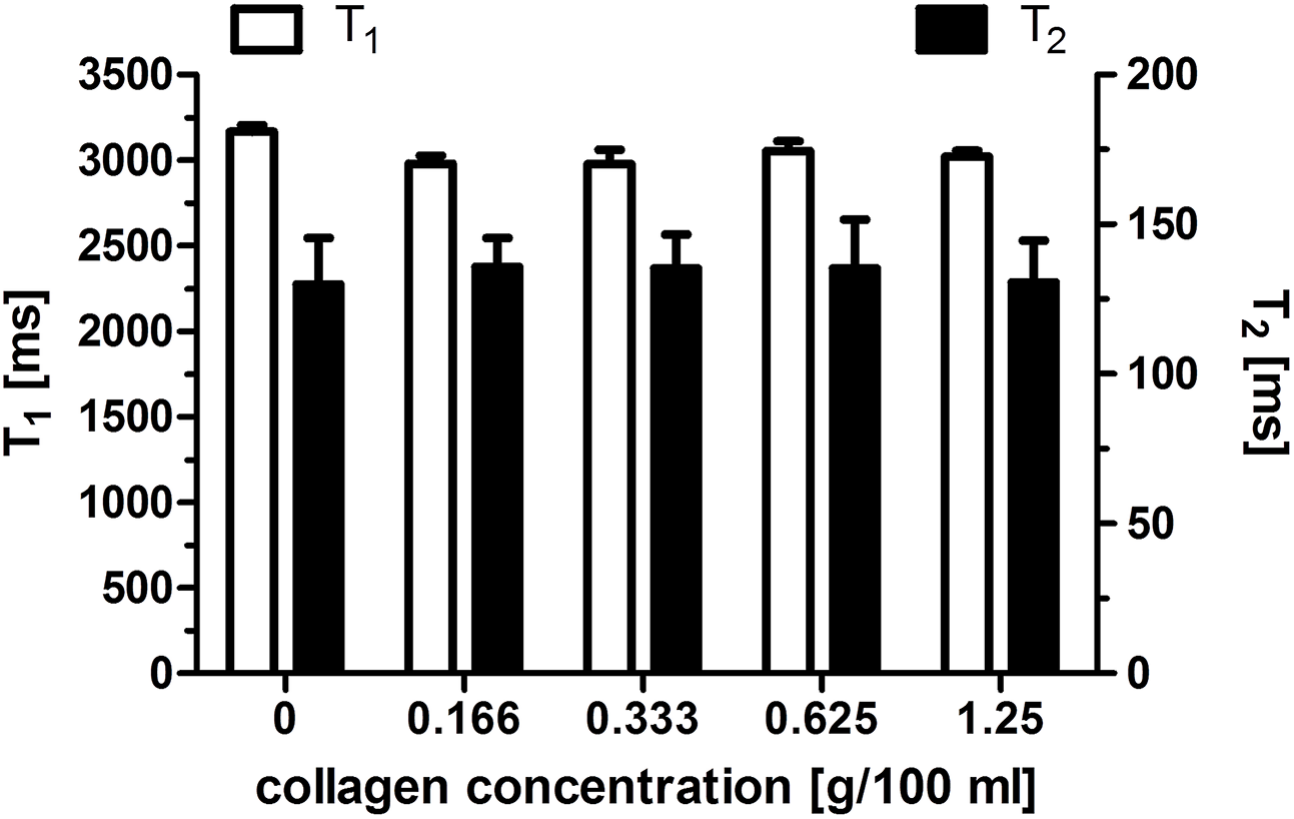
Relaxometry of collagen solutions. This figure shows the absence of significant variation of relaxation time-constants with collagen concentration.

Relaxometry studies performed on collagen solutions did not reveal any significant change of either T_1_ or T_2_ relaxation time with increasing collagen concentration (Fig 3).

### Histology

**Fig 4:**
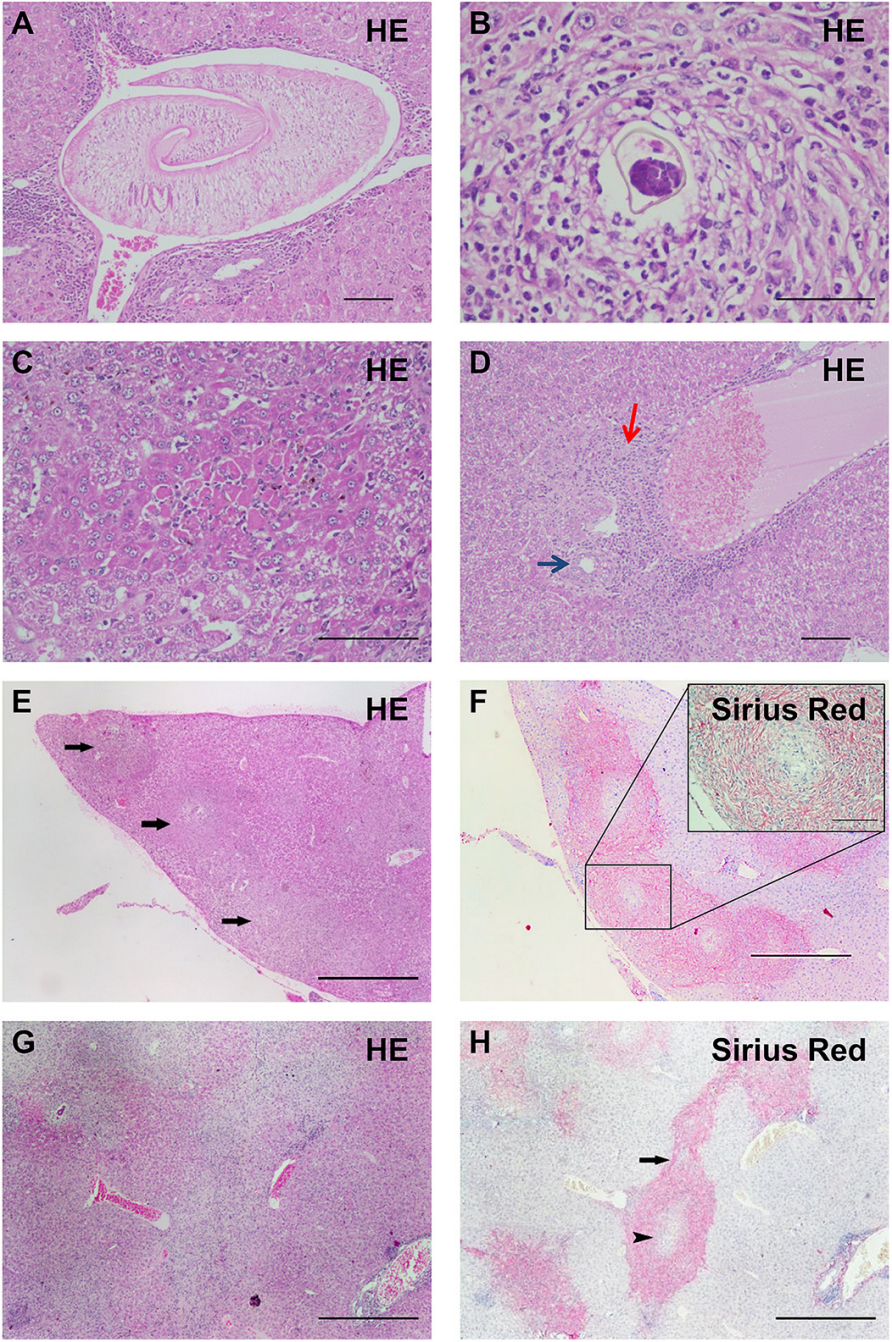
Liver granulomatous pathology and fibrosis. **(A)** Intravascular adult parasite at 6 weeks post infection (HE, scale bar 100 μm). **(B)** to **(H)**: 10 weeks post infection. (**B)** Granuloma centred on one egg (HE, scale bar 50 μm). (**C)** Focal hepatocellular necrosis (HE, scale bar 100 μm). (**D)** Periportal eosinophilic inflammation (red arrow) around a bile duct (blue arrow) (HE, scale bar 100 μm). (**E)** and **(F)** Liver lobe allowing assessment of the extent of granulomatous chronic inflammation and hepatitis (arrows pointing at granulomas) in HE staining **(E)** and portal fibrosis with collagen deposition assessed with Sirius Red staining (**F**) (bar 500 μm). Sirius Red facilitates quantification of extracellular matrix deposition of collagen within granulomas (zoom in **F**, bar 100 μm). (**G)** and **(H**) Intergranulomatous fibrosis in HE staining (**G**) and Sirius Red staining (**H**) (arrowhead pointing at granuloma and arrow at intergranulomatous fibrosis) (scale bar 500 μm). HE = Hematoxylin-Eosin.

Histology showed that adult parasites (Fig 4A) and eggs began to lodge in the liver with minimal inflammation and occasionally with isolated periovular granulomas (S1B Fig) by 6 weeks post infection, corresponding to METAVIR scores ≤ A1F0. All infected animals showed discrete periportal and portal inflammation at 6 weeks post infection but only one of them was considered A1F0. The number of eggs trapped in liver tissue dramatically increased thereafter. By 10 weeks post infection, 4 mice presented with severe portal fibrosis with granulomatous chronic inflammation, corresponding to METAVIR scores of A1F1 to A2F2 (Fig 4B–4H). Eggs were surrounded by a dense population of immune cells, with mild to marked extracellular matrix deposition leading to intergranulomatous fibrosis and fusion of several granulomas replacing some portal spaces (Fig 4E–4H).

In a large number of advanced stage granulomas, pigment accumulation in macrophages (S1C Fig), which appeared negative on the Perls’ stain (S1D Fig), was present. This is highly suggestive of hematin, a degradation product of hemoglobin excreted by the worm [42]. Histology confirmed the absence of lesions in the two remaining mice in which the infection had failed; consequently these mice were excluded from analysis.

**Fig 5:**
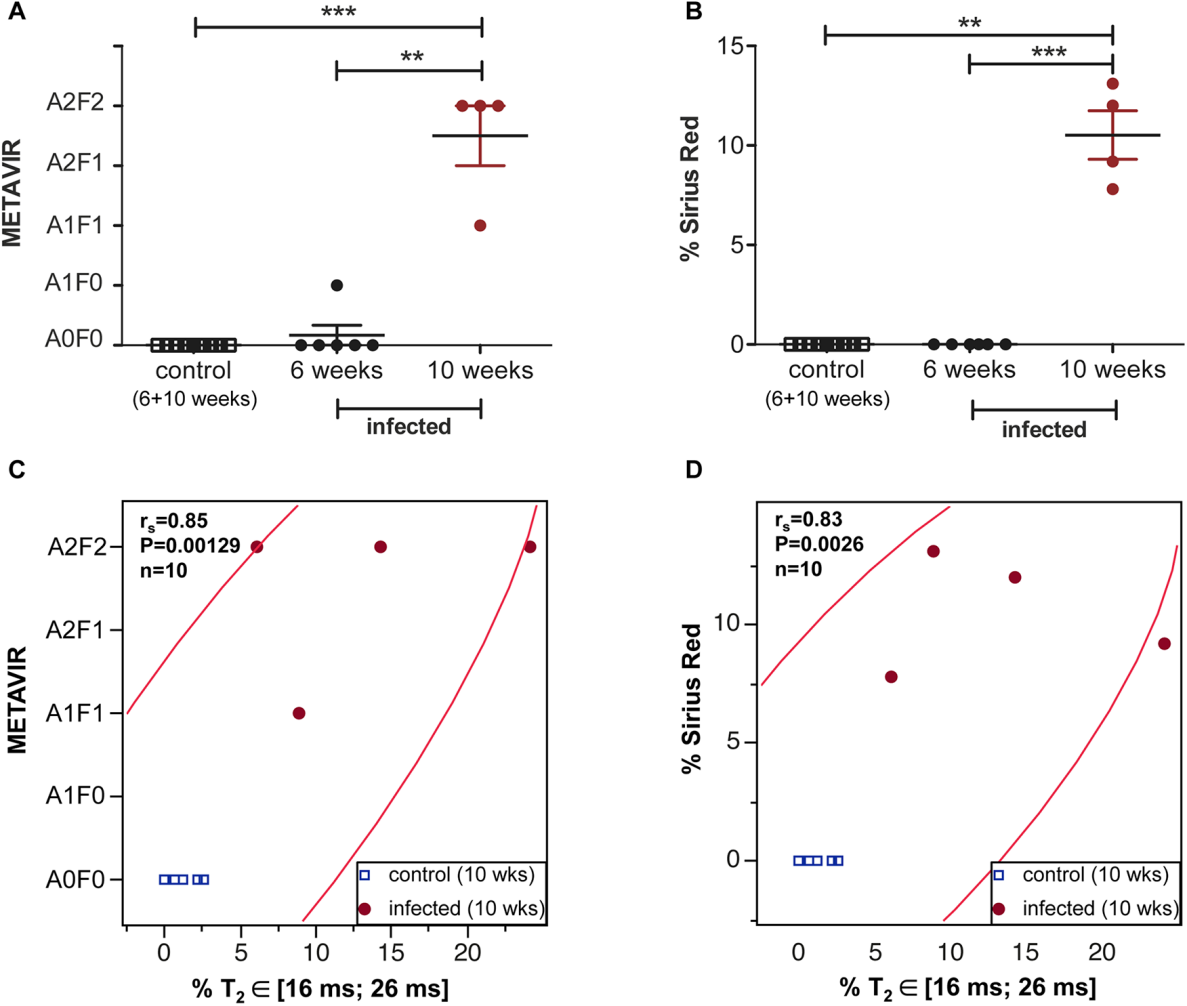
Correlation between relaxometric and histological markers of disease severity. Disease severity as assessed by the METAVIR score **(A)** and the area fraction stained with Sirius Red for collagen **(B)** increases mainly 6 weeks after infection. The area fraction of liver T_2_ values between 16 and 26 ms obtained at 10 weeks after infection correlates with the METAVIR grading (**C**) and fibrosis quantification with Sirius Red staining (**D**). A 95% bivariate normal density ellipse is represented on each scatterplot. (The correlations obtained for the values measured at 6 and 10 weeks are shown in S2A and S2B Fig).

METAVIR score and area fraction of fibrosis stained with Sirius Red increased simultaneously from 6 weeks post infection on (Fig 5A and 5B) and were both significantly correlated with the area fraction of T_2_ values comprised between 16 and 26 ms (Fig 5C and 5D) at 10 weeks after infection. Since control values obtained at 6 and 10 weeks for the METAVIR score or the Sirius Red staining were identical (A0F0, or 0% respectively), they were pooled in the statistical analysis (Fig 5A and 5B). The correlations obtained for the values measured at 6 and 10 weeks are shown in S2A and S2B Fig.

## Discussion

To our knowledge, this is the first time a murine model of schistosomiasis is evaluated by quantitative MRI. Anatomical MR images of *S*. *mansoni* infected mouse liver had already been published, but a systematic follow-up during disease development was not reported [43]. These images were obtained from a mouse infected with subcutaneous injection of 150 *cercariae*, while our model relies on the percutaneous administration of only 30 *cercariae*, replicating the natural route of infection and leading to a similar disease burden obtained with the injection of 150 *cercariae*. The pathological findings obtained with *in vivo* MRI in this model are in accordance with known features of the human disease, confirming its relevance to human disease. Volumetric analyses revealed hepato- and splenomegaly as well as portal vein cross section increase as early as 6 weeks post infection, and reliably detected aggravation of these signs during disease progression. These symptoms are unlikely to be a consequence of hepatic fibrosis, which was undetectable at 6 weeks post infection, but rather of vascular damage and obstruction caused by the schistosome eggs [7,44]. Hepatic tissue alteration and iron accumulation were detected 10 weeks post infection by MRI as T_2_ and T_2_* changes, respectively, and were confirmed by histology.

From a technical point of view, the multiparametric MRI protocol used in this study was sensitive enough to monitor disease severity and identify infection failure in two out of 6 mice. In particular, the correlation with the area fraction of collagen stained with Sirius Red in liver histology shows that the transverse relaxation time T_2_ can be used as a quantitative non-invasive biomarker of hepatic fibrosis.

Few techniques are sensitive to potentially reversible early stages of liver fibrosis. All existing imaging approaches rely on the measurement of parameters that are only indirectly related to liver fibrosis (tissue stiffness, restricted water diffusion). Likewise, relaxation time constants depend on physicochemical properties that are altered during fibrogenesis. In *ex vivo* studies, correlation between transverse relaxation times and fibrosis has been controversial. For example, at 11.75T a T_2_ decrease has been observed in a 3,5-diethoxycarbonyl-1,4-dihydrocollidine induced mouse model of hepatic fibrosis [45]. But in another study at 0.94T a positive correlation between liver T_2_ and degree of fibrosis was observed in a thioacetamide induced mouse model of liver cirrhosis [34]. It is known that the T_2_ is a rather unspecific parameter, influenced by inflammation [34,46], edema, perfusion changes or steatosis [47]. Indeed, free water is characterized by a long T_2_ (hundreds of ms) whereas lipids have much shorter T_2_ values (tens of ms). In the vast majority of cases, severe hepatic fibrosis in human schistosomiasis is not associated with ultrasound detectable steatosis namely the accumulation of triglycerides in hepatocytes, except in patients with obesity or diabetes. Most subjects with severe disease are rather underweight. Moreover, liver metabolic profiling by ^1^H nuclear magnetic resonance spectroscopy revealed reduced levels of lipids in *S*. *mansoni* infected mice [48]. In agreement with previous results [49], our *in vitro* experiments show that collagen at concentrations up to 12.5 g/L does not significantly modify the relaxation times. The range of collagen concentrations tested in our phantom studies corresponds to those measured by biochemical techniques in whole-liver extracts of *S*. *mansoni*-infected mice at 8 weeks after infection with 50 *cercariae (S*. *mansoni* infected mice: 10.3 ±1.60 mg/g of wet liver, control mice: 0.591±1.144 mg/g of wet liver) [50]. However, deposition of collagen (Fig 4D) in the extracellular matrix in living tissue is accompanied by pathophysiological reactions that have a measurable effect on relaxation times [19]. We cannot discard the possibility that other components of the extra-cellular matrix, which accumulate during fibrogenesis, (fibronectin, elastin, proteoglycan, hyaluronan etc…) may contribute to T_2_ changes. Along the same line, it is not excluded that the observed T_2_ increase in the liver of *S*. *mansoni* infected mice is due to the decreased content of hepatic lipids [48], but further investigations are required.

In agreement with other *in vivo* MRI studies in animal models of hepatic fibrosis [35,36] and in humans [51], at 10 weeks post infection we observed a T_2_ increase superior to 14% compared to normal liver tissue. Although the T_2_ increase might be related to inflammation and edema, the hepatic T_2_ values were not yet increased 6 weeks after infection (10.4 ± 2.4 *versus* 10.7 ± 2.5 ms in infected *versus* control mice) although histology revealed a discrete (A0 –A1) portal inflammatory reaction at this stage of the disease, without any sign of fibrosis.

In contrast to previous works that assessed the alteration of the average T_2_ value in the presence of liver fibrosis [34,35,36,45], we quantified the area fraction of altered T_2_ on T_2_ maps of liver tissue. This measure was not performed in previous studies, despite a non-uniform distribution of fibrosis in most cases and attempts to correlate quantitative MRI parameters to the area fraction of fibrosis observed in histology. Our results demonstrate that despite the lack of specificity of the T_2_ relaxation time, the area fraction of increased T_2_ in the liver is significantly correlated with the area fraction affected by fibrosis. The area fraction of increased T_2_ enabled us to differentiate between early and intermediate fibrosis stages (i.e. stages ≤ F2).

### Study limitations

This exploratory study on a small number of animals aimed at evaluating a non-invasive MRI method for the assessment of liver fibrosis progression. A limitation of this study is that different animal groups were used at the last two imaging time points, due to the fact that a validation by histology was mandatory. A longitudinal study on the same group of animals up to a time point beyond 10 weeks post infection would be desirable.

### Advantages

For disease monitoring and treatment evaluation in small animals T_2_ mapping is easy to implement and less challenging than transient elastography or MRE. Indeed, this technique does not require the use of additional equipment such as vibrators and sequences for relaxometry are readily available on standard pre-clinical MRI systems.

Magnetic resonance relaxometry has the advantage of being quantitative without any contrast agent injection that could interfere with biological parameters and alter disease development. However, administration of a commercially available nonspecific extracellular contrast agent such as Gd-DOTA enhanced the lesions, demonstrating increased vessel permeability.

The quantitative T_2_ and T_2_* maps performed in this study allow further optimization of image weighting parameters for future studies on schistosomiasis.

### Conclusion

T_2_ mapping is a quantitative and non-invasive marker of non-steatotic liver fibrosis with sensitivity close to that of histology even at early stages.

This multiparametric and quantitative MRI approach can monitor hepatic and splenic disease progression and assess liver fibrosis non-invasively. In preclinical studies and in settings were MRI facilities are readily available, this objective quantitative imaging biomarker may be used to monitor response to therapy in diseases involving hepatic fibrosis.

## Supporting Information

S1 Fig Liver granulomatous pathology and fibrosis. **A:** Morphometrical analysis of fibrosis (Sirius Red, scale bar 500 μm). **B:** Periovular granuloma with eosinophilic and lymphoplasmocytic infiltration without collagen deposition 6 weeks post infection (HE, scale bar 50 μm). **C:** Granuloma with multinucleate giant cells (arrows) containing brown pigment 10 weeks post infection (HE, scale bar 50 μm). **D:** Negative Perls’ reaction confirms Hematin deposits within a granuloma (Perls’ stain, scale bar 100 μm). HE = Hematoxylin-Eosin.
(TIF)

S2 Fig Correlation between relaxometric and histological markers of disease severity for values obtained at 6 weeks and 10 weeks after infection. The area fraction of liver T_2_ values between 16 and 26 ms obtained at 6 and 10 weeks after infection correlates with the METAVIR grading (A) and fibrosis quantification with Sirius Red staining (B). A 95% bivariate normal density ellipse is represented on each scatterplot.
(TIF)
