# Correction: *In Vivo* MRI Assessment of Hepatic and Splenic Disease in a Murine Model of Schistosmiasis

**DOI:** 10.1371/journal.pntd.0004174

**Published:** 2015-10-16

**Authors:** Brice Masi, Teodora-Adriana Perles-Barbacaru, Caroline Laprie, Helia Dessein, Monique Bernard, Alain Dessein, Angèle Viola

The word “Schistosomiasis” is misspelled in the article title. The correct title is: *In Vivo* MRI Assessment of Hepatic and Splenic Disease in a Murine Model of Schistosomiasis. The correct citation is: Masi B, Perles-Barbacaru T-A, Laprie C, Dessein H, Bernard M, Dessein A, et al. (2015) *In Vivo* MRI Assessment of Hepatic and Splenic Disease in a Murine Model of Schistosomiasis. PLoS Negl Trop Dis 9(9): e0004036. doi:10.1371/journal.pntd.0004036
