# Supplementary figures and images for: In Vivo MRI Assessment of Hepatic and Splenic Disease in a Murine Model of Schistosmiasis
Source: PLoS Negl Trop Dis. 2015 Sep 22;9(9):e0004036. doi: 10.1371/journal.pntd.0004036 (PMC4578925; doi:10.1371/journal.pntd.0004036)

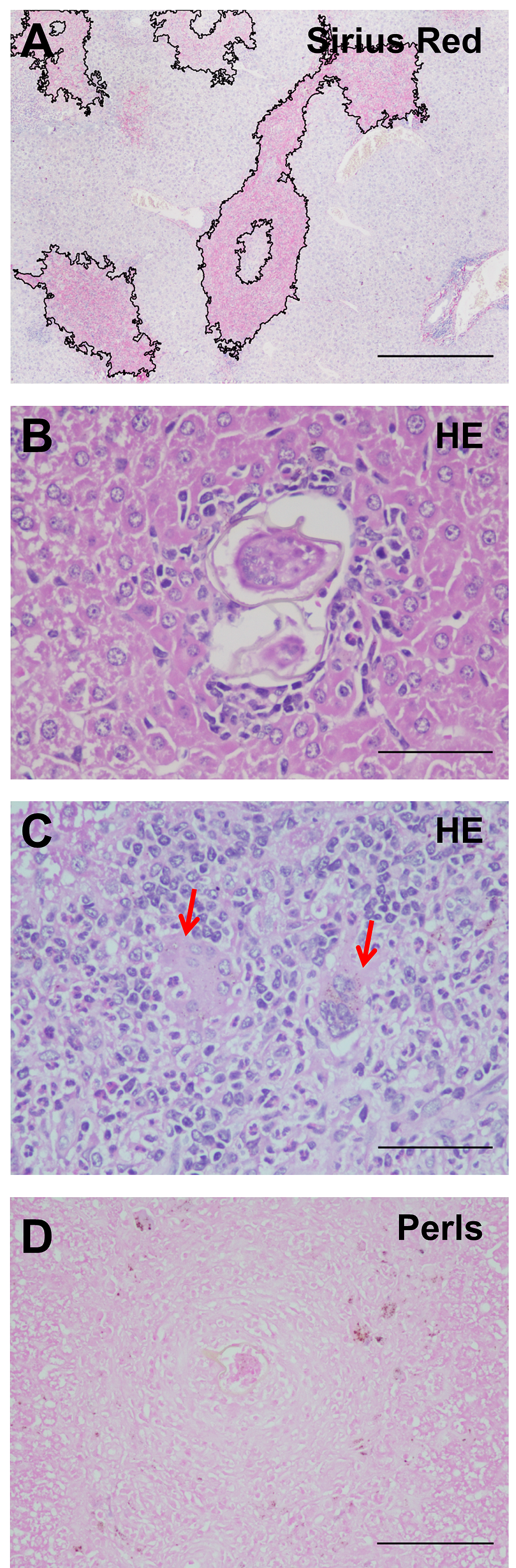

Supplement: S1 Fig — A: Morphometrical analysis of fibrosis (Sirius Red, scale bar 500 μm). B: Periovular granuloma with eosinophilic and lymphoplasmocytic infiltration without collagen deposition 6 weeks post infection (HE, scale bar 50 μm). C: Granuloma with multinucleate giant cells (arrows) containing brown pigment 10 weeks post infection (HE, scale bar 50 μm). D: Negative Perls’ reaction confirms Hematin deposits within a granuloma (Perls’ stain, scale bar 100 μm). HE = Hematoxylin-Eosin. (TIF) [file pntd.0004036.s001.tif]

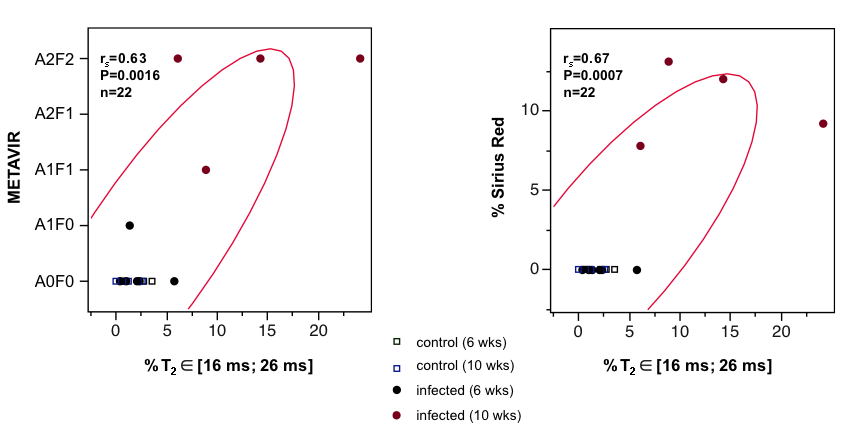

Supplement: S2 Fig — The area fraction of liver T2 values between 16 and 26 ms obtained at 6 and 10 weeks after infection correlates with the METAVIR grading (A) and fibrosis quantification with Sirius Red staining (B). A 95% bivariate normal density ellipse is represented on each scatterplot. (TIF) [file pntd.0004036.s002.tif]
